# Effect of Vojta technique on improvement in respiratory function and neuromuscular impairment in infants with preterm: a systematic review protocol

**DOI:** 10.1186/s13643-025-02988-9

**Published:** 2025-11-17

**Authors:** Sharath Hullumani, Irshad Qureshi, Raghuveer Raghumahanti, Sakshi Desai, Neha Brahmane, Pratiksha Warghat

**Affiliations:** 1 Department of Paediatric and Neonatal Physiotherapy, Ravi Nair Physiotherapy College, Datta Meghe Institute of Higher Education and Research (DU) Sawangi Meghe, Maharashtra, Wardha India; 2 Department of Neuro Physiotherapy, Ravi Nair Physiotherapy College, Datta Meghe Institute of Higher Education and Research (DU) Sawangi Meghe, Maharashtra, Wardha India

**Keywords:** Chest physiotherapy, Newborns, Preterm, Infants

## Abstract

This systematic review protocol introduces the rationale to investigate how well the Vojta approach works to improve respiratory function and lessen neuromuscular deficits in premature newborns. Innovative approaches like the Vojta technique may offer a possible therapeutic path for preterm newborns, who frequently suffer significant obstacles in their early development. We outline a rigorous methodology for this systematic review. A comprehensive search strategy will be implemented across various databases to identify relevant studies. This systematic review will encompass randomized controlled trials through electronic and manual searches. Electronic searches will be carried out in databases such as PubMed, Medline, Scopus, and Web of Science. The search will span articles published from 2000 to 2025; predefined eligibility criteria involve participants who were premature babies (those born before 37 weeks gestation). Exposure includes participants diagnosed with any other diagnosis that does not include NRDS. Outcome measures focus on oxygen saturation levels (SpO₂), arterial blood gas (ABG) analysis (e.g., PaO₂, PaCO₂, and pH), respiratory rate (breaths per minute), and lung compliance (static or dynamic) will all be used to quantify respiratory function. Two independent reviewers will conduct study selection, data extraction, and risk of bias assessment. The primary outcomes encompassing SpO₂, ABG, Ballard, and APGAR will be systematically analysed.

**Dissemination and ethics** As no primary data will be collected, ethical approval is not required. The findings will be presented at relevant conferences and published in a peer-reviewed journal.

**Systematic review registration** PROSPERO CRD420250643755.

**Supplementary Information:**

The online version contains supplementary material available at 10.1186/s13643-025-02988-9.

## Introduction

Neonatal health is severely hampered by preterm birth, which is defined as delivery before 37 full weeks of pregnancy, especially in the areas of respiratory function and neuromuscular control [1, 2]. Premature babies are more likely to experience problems such as bronchopulmonary dysplasia and delayed motor development, which can negatively impact their general development and quality of life for a long time [2]. Numerous therapy approaches have been investigated in response to these difficulties. Among these, the Vojta technique, a neurophysiological method initially created to treat motor problems, has gained attention as a possible way to enhance respiratory mechanics and neuromuscular coordination by inducing innate reflex locomotion. Specialized therapies are frequently needed to assist the respiratory and neuromuscular development of preterm newborns. High-flow nasal cannula (HFNC), nasal intermittent positive pressure ventilation (NIPPV), and nasal continuous positive airway pressure (nCPAP) are respiratory support techniques that help preserve airway patency, enhance oxygenation, and lessen the need for invasive ventilation. Infants with respiratory distress syndrome are often treated with surfactant therapy to improve lung compliance and lower alveolar surface tension. Other helpful techniques include kangaroo mother care, which encourages thermoregulation, respiratory stability, and parent-infant bonding, and prone positioning, which stabilizes breathing patterns and lessens apnea episodes [3] together, these treatments seek to maximize functional results and support preterm infants’ developmental progress.

By activating reflex patterns through particular pressure spots and posture, the Vojta technique may improve muscle tone and coordination in newborns who are at risk [4]. The Vojta approach has been shown to be effective in populations with neurological deficits, but its use in preterm newborns has not received enough attention [5, 6]. Although preliminary research indicates that early intervention with this approach may result in improvements in neuromuscular function and respiratory parameters, a thorough assessment of the data is required to support these assertions [7, 8].

The purpose of this systematic review protocol is to thoroughly evaluate the body of research on the application of the Vojta technique to premature newborns. In order to inform clinical practice and direct future research endeavors, this review attempts to ascertain whether the Vojta method can function as an effective intervention for improving respiratory performance and mitigating neuromuscular impairments in this high-risk population by synthesizing current research findings [9, 10].

### Research question

What impact does the Vojta technique have on preterm infants’ respiratory function and neuromuscular impairment?

## Methods

PROSPERO Registration Number: CRD420250643755; https://www.crd.york.ac.uk/PROSPERO/view/CRD420250643755.

### Eligibility criteria

1. Population (P): Premature babies (those born before 37 weeks gestation).
  Infants with neuromuscular impairments or respiratory function problems that are frequently linked to prematurity, including as motor function delays, persistent lung illness, or respiratory distress
2. Intervention (I): A physiotherapeutic approach created by Dr. Vaclav Vojta with the goal of improving motor functions by stimulating particular bodily reflex sites, which are thought to trigger innate motor responses and enhance neuromuscular coordination.
3. Comparison (C): Absence of treatment, comparative group is necessary for studies and it could be a placebo.
  This usually covers the standard medical and supportive care given to premature babies, which may include respiratory support techniques, basic physical therapy, and general pediatric care. Commonly used interventions for similar issues in premature newborns include Bobath therapy, traditional physical therapy, or more recent physiotherapeutic approaches.
4. Outcome measures (O):
  Oxygen saturation levels (SpO₂), arterial blood gas (ABG) analysis (e.g., PaO₂, PaCO₂, and pH), respiratory rate (breaths per minute), and lung compliance (static or dynamic) will all be used to quantify respiratory function.
  Length of hospital stay.
  The length of respiratory support, including mechanical ventilation, CPAP, or oxygen therapy, shall be documented from the start of the intervention to the end of respiratory support.
  Enhancement of respiratory performance: Clinical evaluations such as oxygen saturation levels, pulmonary function tests (PFTs), and the requirement for respiratory support (such as mechanical ventilation) can be used to gauge respiratory performance, decrease in neuromuscular impairment as measured by the Ballard score and APGAR.
5. Study design: The study will be focused on observational studies, experimental studies, and randomized controlled trials (RCT).
6. Language: Only English-language research or studies with available translations will be deemed eligible for this study due to translation restrictions.
7. Publication date: Studies published from 2000 to 2025 will be considered.
8. Publication type: Both published article and grey literature.

### Information sources

To find papers that are pertinent to our study issue, we will use both manual and electronic searches. Four databases—PubMed, Embase, CINAHL, and the Cochrane Library—will be searched electronically (from inspection to December 2024). Although it is acknowledged as a search engine rather than a formal database, the first 50 pages of Google Scholar pages will be used to supplement the search. Depending on the needs of each database, our search algorithms will blend free texts and medical subject headings (MeSH). Due to the low quality of the evidence, individual case reports, case series, and expert views will also not be included in the data extraction process. Articles that are not published or in full text will also not be included.

### Selection process and data collection process

(S.H. and I.Q.), two calibrated and independent reviewers, will pick the study in two stages. The first step involves screening abstracts and titles for alignment with the inclusion criteria and research question. To decide on final inclusion, the full texts of possibly pertinent research are examined in the second stage. This procedure will be guided by the inclusion and exclusion criteria. In phase 1, the titles and abstracts of the chosen papers will be read by the two reviewers, S.H. and I.Q. The entire texts of the previously included papers will be read by the same reviewers (S.H. and I.Q.) in phase two. The selection criteria will be the same as those from the first round. A third reviewer (R.R.) will make the decision if there is a dispute.

### Data items and extraction

Participant characteristics will include baseline characteristics like gestational age (mean age and age range), gender distribution, and inclusion and exclusion criteria, respiratory function, Ballard score, and APGAR among other pertinent demographic or clinical data. Details of the intervention will include the type of physiotherapy intervention (e.g., Vojta, chest physiotherapy), its duration, frequency, intensity, and mode of delivery. Information about the sort of control or comparative interventions (such as a placebo, standard care, or no intervention), as well as its length, frequency, and intensity, will be gathered for comparators. Respiratory function and neuromuscular impairment will be the main focus of outcome measures. APGAR and Ballard Score for Neuromuscular Impairment, lung compliance (static or dynamic), oxygen saturation levels (SpO₂), and arterial blood gas (ABG) analysis (e.g., PaO₂, PaCO₂, and pH) will be used to evaluate the respiratory function and any adverse effects reported. The study design (such as an observational study or randomized controlled trial), sample size, funding sources to evaluate potential bias, and any disclosed conflicts of interest by the study authors are all examples of study features. To ensure consistent and accurate data extraction and analysis across all included research, pre-planned data assumptions and simplifications will be used when needed [11, 12].

### Outcomes

Improvements in respiratory function and neuromuscular impairment are the main goals for this review. Oxygen saturation levels (SpO₂), arterial blood gas (ABG) analysis (e.g., PaO₂, PaCO₂, and pH), respiratory rate (breaths per minute), and lung compliance (static or dynamic) will all be used to quantify respiratory function. These measures will be evaluated using pulmonary function tests or ventilator settings. Pre-intervention, just after the intervention, and at follow-up times like 24 to 72 h and 1 week will all have these measurements taken. Validated scoring methods, such as the APGAR and Ballard Score, will be used to assess neuromuscular impairment. Data will be collected at baseline, right after the intervention, and at follow-ups. The length of hospital stay and the duration of respiratory assistance are examples of secondary outcomes. The length of respiratory support, including mechanical ventilation, CPAP, or oxygen therapy, shall be documented from the start of the intervention to the end of respiratory support. This duration will be expressed in hours or days.

### Assessment of risk of bias in included studies

The risk of bias in the included studies will be evaluated by two independent reviewers, S.H. and I.Q. Depending on the sort of investigation, different tools will be employed: ROBINS-I for non-randomized intervention studies, ROBINS-E for observational research, and RoB 2.0 (www.riskofbias.info) for randomized clinical trials. A third reviewer will resolve disagreements over the evaluation (R.R.). The authors will talk about the tool and set the settings for its assessment before using it. A calibration between the evaluators will then be conducted as well. Each tool’s suggested presentation of the results will be used. The Robvis website will be used to make figures (https://www.riskofbias.info/welcome/robvis-visualization-tool). If there are enough studies (typically more than 10) to address publication bias, a funnel plot will be employed. We will use either Egger’s or Begg’s test to statistically analyze the funnel plot’s asymmetry [11, 12].

### Data synthesis

Standardized mean differences (SMD) or relative risks (rrs) with 95% confidence intervals (cis) will be used to measure the effectiveness of treatment for preterm infants in order to assess primary outcomes like respiratory function and for neuromuscular impairment. A narrative approach will be the main method used for data synthesis due to the expected heterogeneity among research. Key outcomes, types of interventions, and their reported benefits on respiratory function and for neuromuscular impairment will be highlighted in a descriptive summary of the findings from the included studies. To improve clarity, pictorial representations will be added to a narrative summary. To evaluate variations in the effects of the intervention across participant demographics or intervention attributes, subgroup analyses will be carried out [11–13].

### Confidence in cumulative evidence

Using the GRADE (Grading of Recommendations, Assessment, Development, and Evaluation) method, this systematic review will be assessed. When it comes to handling neonates with respiratory distress syndrome, the GRADE approach may be able to perform a thorough evaluation of the presence of evidence supporting the efficacy of physiotherapy treatments.

## Discussion

The main conclusions of the systematic review would be summarized first, with special attention to the effects of the Vojta technique on preterm infants’ neuromuscular development and respiratory health. It would place the findings into the broader body of research by contrasting these findings with previous research to identify any similarities or differences. The review has important therapeutic ramifications; if the method is shown to be successful, it may be suggested for wider clinical use and could eventually be incorporated into routine preterm infant care regimens. The assessment also emphasizes the need for more research, highlighting certain areas that require more investigation to improve the Vojta technique’s application and confirm its safety and effectiveness. Maintaining scientific integrity requires acknowledging the review’s limitations, including potential biases and the diversity of study designs. The conclusion concludes by restating the Vojta technique’s ability to improve the care of preterm newborns and highlighting its implications for educational programs and legislation aimed at educating healthcare workers on its use.

### Strength and limitations

This study was designed using the Preferred Reporting Items for Systematic Review and Meta-Analysis Protocols (PRISMA-P) and the Meta-Analyses and Systematic Reviews of Observational Studies (MOOSE) standards. A third reviewer will mediate any disagreements over data extraction and study inclusion after two independent reviewers select the research. Due to translation limitations, the literature will only include English-only texts, which will reduce the overall amount of data that can be extracted. If there was not enough data available for extraction and analysis, the study will outline any knowledge gaps and provide guidance for future research in these areas.

## Supplementary Information

Additional file 1. PRISMA checklist

## Data Availability

No data are available.

## References

[1] Rodriguez Gonzalez P, Perez-Cabezas V, Chamorro- Moriana G, Ruiz Molinero C, Vazquez-Casares AM, Gonzalez- Medina G. Effectiveness of oral sensory-motor stimulation in premature infants in the Neonatal Intensive Care Unit (NICU) systematic review. Children (Basel). 2021;8:758. 10.3390/children8090758. 34572190 10.3390/children8090758 PMC8465336

[2] Sweeney JK, Heriza CB, Blanchard Y, Dusing SC. Neonatal physical therapy. Part II: practice frameworks and evidence- based practice guidelines. Pediatr Phys Ther. 2010;22:2–16. 20142700 10.1097/PEP.0b013e3181cdba43

[3] Igual Blasco A, Piñero Peñalver J, Fernández-Rego J, Torró-Ferrero G, Pérez-López J. Effects of chest physiotherapy in preterm infants with respiratory distress syndrome: a systematic review. Healthcare. 2023;11(8):1091. 10.3390/healthcare11081091. 37107923 10.3390/healthcare11081091 PMC10137956

[4] Llamas-Ramos R, Sánchez-González JL, Alvarado-Omenat JJ, Rodríguez-Pérez V, Llamas-Ramos I. Brain electrical activity and oxygenation by reflex locomotion therapy and massage in preterm and term infants. A protocol study. NeuroImage. 2024;298:1–6. 10.1016/j.neuroimage.2024.120765.

[5] Trafalska A, Paprocka-Borowicz M. The role of the Vojta method in diagnosing and enhancing motor skills in preterm infants: a prospective open-label controlled study. Med Sci Monit. 2025;31:e945495. 10.12659/MSM.945495. 40156119 10.12659/MSM.945495 PMC11963827

[6] Sánchez-González JL, Sanz-Esteban I, Menéndez-Pardiñas M, Navarro-López V, Sanz-Mengíbar JM. Critical review of the evidence for Vojta therapy: a systematic review and meta-analysis. Front Neurol. 2024;15:1391448. 10.3389/fneur.2024.1391448. 38711552 10.3389/fneur.2024.1391448 PMC11070493

[7] Igual Blasco A, Piñero Peñalver J, Fernández-Rego FJ, Torró-Ferrero G, Pérez-López J. Effects of chest physiotherapy in preterm infants with respiratory distress syndrome: a systematic review. Healthcare. 2023;11(8):1091. 10.3390/healthcare11081091. 37107923 10.3390/healthcare11081091 PMC10137956

[8] Gomez-Conesa A, Rego FF, Arenas JA Vojta therapy in the reduction of perinatal risk in preterm infants with respiratory distress syndrome and bronchopulmonary dysplasia. Physiotherapy. 2016;102. 10.1016/j.physio.2016.10.242.

[9] Elmoneim MA, Mohamed HA, Awad A, et al. Effect of tactile/kinesthetic massage therapy on growth and body composition of preterm infants. Eur J Pediatr. 2021;180(1):207–15. 32666281 10.1007/s00431-020-03738-w

[10] Van den Hoogen A, Teunis CJ, Shellhaas RA, Pillen S, Benders M, Dudink J. How to improve sleep in a neonatal intensive care unit: a systematic review. Early Hum Dev. 2017;113:78–86. 28720290 10.1016/j.earlhumdev.2017.07.002

[11] McNair C, Chirinian N, Uleryk E, Stevens B, McAllister M, Franck LS, et al. Effectiveness of parental education about pain in the neonatal period on knowledge, attitudes, and practices: a systematic review and meta-analysis. Paediatr Child Health. 2022;27(8):454–63. 10.1093/pch/pxac050. 36583071 10.1093/pch/pxac050 PMC9792286

[12] Higgins JPT et al. Cochrane handbook for systematic reviews of interventions version 6.0 (updated July 2019). Cochrane, 2019. www.training.cochrane.org/handbook.

[13] Munn Z, Stern C, Aromataris E, Lockwood C, Jordan Z. What kind of systematic review should I conduct? A proposed typology and guidance for systematic reviewers in the medical and health sciences. BMC Med Res Methodol. 2018;18(5):pmid:29316881. 10.1186/s12874-017-0468-4 PMC5761190 29316881

